# Health-related quality of life following minimally invasive totally endoscopic mitral valve surgery

**DOI:** 10.1186/s13019-020-01242-8

**Published:** 2020-07-28

**Authors:** Ling-chen Huang, Dao-zhong Chen, Liang-wan Chen, Qi-chen Xu, Zi-he Zheng, Xiao-fu Dai

**Affiliations:** Department of Cardiovascular Surgery, Union Hospital, Fujian Medical University, Fuzhou, 350001 People’s Republic of China

**Keywords:** Minimally invasive, Quality of life, Mitral valve surgery, SF-36

## Abstract

**Background:**

To compare the impact of two different types of mitral valve surgery on health-related quality of life, we conducted a retrospective study comparing modified totally endoscopic mitral valve surgery with median sternotomy mitral valve surgery.

**Methods:**

A total of 163 patients who underwent mitral valve surgery at our institution between January 1, 2019 and December 31, 2019 were enrolled. For these 163 patients, mitral valve surgery was performed using either a modified totally endoscopic approach or median sternotomy approach. We used the numerical rating scale and the Scar Cosmesis Assessment and Rating Scale to measure pain intensity and the aesthetic appearance of the surgical incision and used the MOS 36-item Short-Form Health Survey to assess health-related quality of life.

**Results:**

Seventy-eight patients underwent the modified totally endoscopic mitral valve surgery, and eighty-five patients underwent the median sternotomy mitral valve surgery. The two groups of patients were similar in terms of demographics and echocardiography findings. The number of bioprosthetic valve replacements performed was significantly higher in the totally endoscopic group than in the median sternotomy group (*p* = 0.04), whereas the subvalvular apparatus was less preserved in only 33 cases in the totally endoscopic group (*p* = 0.01). The rate of postoperative adverse events was similar between the two groups. The pain was mild and aesthetic appearance was significantly better in the totally endoscopic approach group than in the sternotomy approach group. Significant differences in the scores for the bodily pain and mental health subscales of the MOS 36-item Short-Form Health Survey were found between the two groups.

**Conclusions:**

Compared with median sternotomy mitral valve surgery, totally endoscopic mitral valve surgery has an equally good treatment effect, improving patient’s health-related quality of life with a better cosmetic appearance and a lower pain intensity. Our study suggested that the totally endoscopic approach is superior to the median sternotomy approach in terms of pain intensity, aesthetic appearance and health-related quality of life.

## Introduction

Minimally invasive mitral valve surgery (MIMVS) has been performed in clinical practice since Cosgrove [1] and Cohn [2] performed the first minimally invasive valve surgery in 1996, and Carpentier [3] and Chitwood [4] subsequently performed video-assisted mitral valve surgery. In China, due to advancements in technology and a significant increase in the demand for minimally invasive approaches from patients, an increasing number of centres are choosing minimally invasive approaches to mitral valve repair and replacement. In recent years, the totally endoscopic approach has become known as a commonly performed and safe technique for mitral valve surgery in our institution. Due to improvements in relevant surgical techniques, the mortality and morbidity rates of these approaches are the same as those of the median sternotomy approach [5, 6]. We reviewed the literature and found that few studies have focused on totally endoscopic mitral valve surgery in terms of pain intensity, cosmetic appearance and health-related quality of life (HRQoL). Therefore, we conducted a retrospective cohort study including 163 patients who had undergone mitral valve surgery using either the modified totally endoscopic approach or median sternotomy approach at our institution and compared the aesthetic appearance of the surgical incision and the HRQoL and pain intensity of these patients. In this study, we performed the modified minimally invasive totally endoscopic approach for mitral valve surgery with cardiopulmonary bypass through the femoral artery and femoral vein only with a single two-stage femoral venous cannula.

## Materials and methods

### Patient selection and data collection

A total of 163 consecutive patients who underwent mitral valve surgery at our institution from January 1, 2019 to December 31, 2019 were enrolled. Mitral valve surgery was performed using either a modified totally endoscopic approach or median sternotomy approach. All patients either returned to the outpatient department for follow-up visits or were contacted by smartphone to confirm all the data that were collected. All patients were followed up, and all the data were available in the patient files. All participants were requested to complete the relevant questionnaires in different ways.

This was a retrospective cohort study that included 163 patients and reported severe events according to the guidelines for reporting mortality and morbidity after cardiac valve interventions that were published in 2008 [7]. The inclusion and exclusion criteria were discussed with and modified according to the expert’s opinion [8]. The inclusion criteria were as follows: (1) primary mitral valve disease; (2) no prior right thoracic surgery; (3) no hearing disorders; (4) complete a whole-course follow-up. The exclusion criteria were as follows: (1) the inability to complete a routine examination; (2) the inability to finish the questionnaires; (3) significant peripheral vascular disease; (4) severe cardiac insufficiency; (5) severe pectus excavatum and kyphoscoliosis; (6) additional aortic valve regurgitation and coronary artery disease requiring surgical interventions.

### Surgical technique

#### Anaesthesia and surgical preparation

The anaesthesia protocols for the two different approaches to mitral valve surgery were generally the same, but the minimally invasive approach relied more on perfusion and anaesthesia techniques. We preferred to have patients intubated with a double-lumen endotracheal tube or a single-lumen endotracheal tube with a bronchial blocker to deflate the right lung during surgery. Transoesophageal echocardiography (TEE) is of great importance for cannulation and cannula placement. The radial or brachial arterial pressure was monitored. A non-invasive finger pulse oximeter was placed in the right arm to monitor the oxygen saturation level.

Patients undergoing right minithoracotomy were placed in a supine position, with a pillow under the right scapula to slightly elevate the right hemithorax. The right elbow was bent, and the right forearm was immobilized on the table to expand the axillary space and allow access to the anterior axillary line. Defibrillator pads were placed in the standard positions outside of the operative field.

#### Surgical incisions

**Fig. 1:**
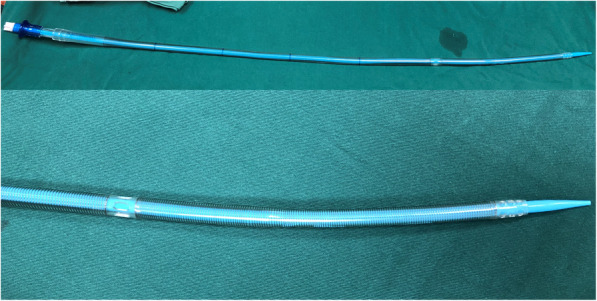
A single two-stage femoral venous cannula

A longitudinal incision was made along the vertical direction of the inguinal ligament to expose the femoral artery and femoral vein, and then, cannulation of the femoral artery and vein was performed with a purse-string suture made of 5–0 polypropylene. After full heparinization [activated clotting time (ACT) > 480 s], the modified Seldinger technique was performed, with an 18-gauge needle and a guide wire [Radifocus® Guidewire M (.35 in., 260 mm), Terumo®] to gain access to the femoral vein. Under TEE guidance, two distal perforated sections of a single two-stage femoral venous cannula [femoral venous cannulae (22 to 30 Fr), Kangxin Medical Instruments Co. Ltd.] were then correctly positioned into both venae cavae (Fig. 1). An arterial cannula was installed through the right femoral artery. The amount of venous drainage from the vena cava was often sufficient when the cannula was placed properly and vacuum-assisted venous drainage was performed [9, 10].

**Fig. 2:**
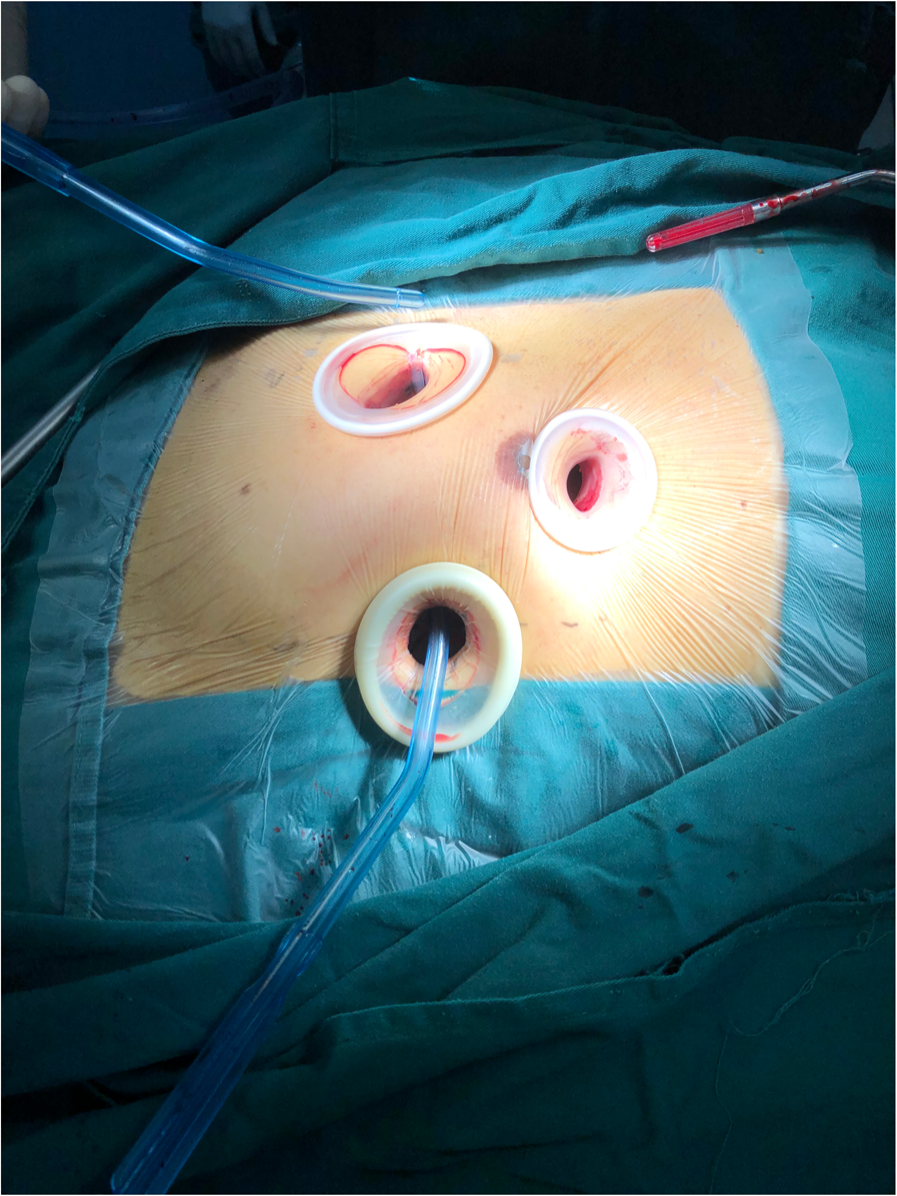
Surgical incisions for totally endoscopic mitral valve surgery

The modified endoscopic approach was performed via endoscopic right minithoracotomy. The primary incision was a 2–4 cm longitudinal incision at the axillary midline in the fourth or fifth intercostal space (usually fourth), depending on the position of the hilum of the right lung on the chest film. We used soft tissue retractors to protect the incision without fracturing the rib cage [WOUND PROTECTORS RETRACTORS, Kangxin Medical Instruments Co. Ltd.]. The primary incision was made to place the thoracoscope, the left heart venting catheter, cardioplegic needle, CO2 line, caval tapes and transthoracic cross-clamp. We used a Chitwood aortic clamp for transthoracic aortic occlusion [11]. Two additional thoracic ports measuring approximately 2–4 cm were installed in the secondary and fifth intercostal spaces for surgical manipulation and insertion of the valve prosthesis (Fig. 2).

#### Surgical process

**Fig. 3:**
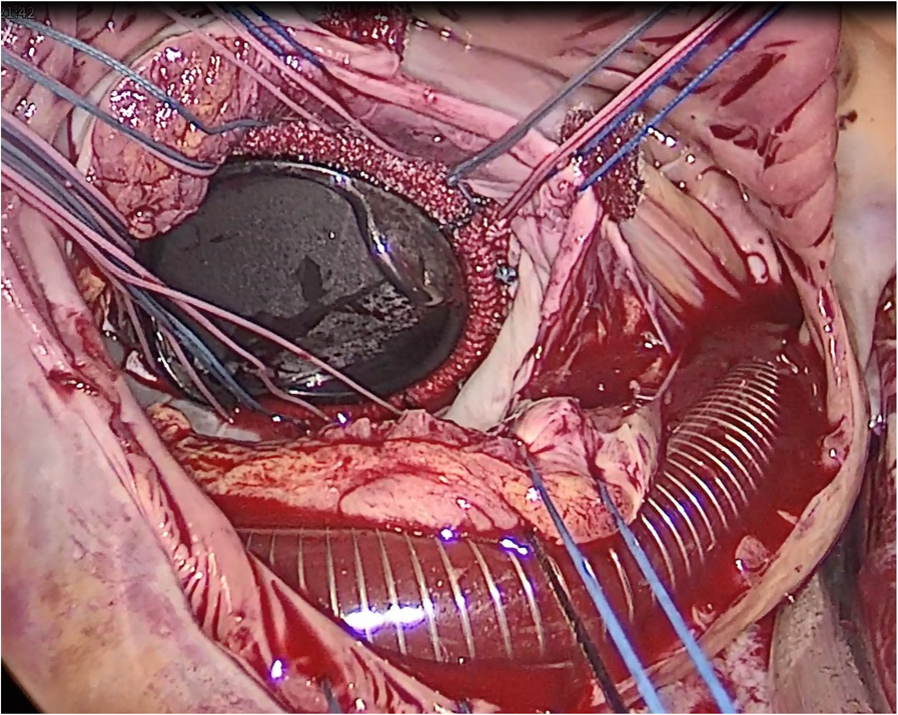
Intraoperative view of the patient with the mechanical valve installed, and correctly position of the single two-stage femoral venous cannula

The anterior pericardium was opened as close to the sternum as possible to create a large flap. The flap was retracted by stay sutures that were inserted in the primary incision, and they held the lung back, creating a large cavity for operation. When full bypass flows and moderate hypothermia were achieved, caval tapes were used to secure the vena cava, and then, the Chitwood clamp was used to occlude the ascending aorta. Then, antegrade HTK solution was administered, and the right atrium was opened. Subsequently, stay sutures were used to retract the margins of the right atrium and were fixed on the chest wall. Then, sutures were used to retract and place the femoral venous cannula in the correct position until we could see the atrial septum (Fig. 3). The left atrium was accessed through the atrial septum, and two groups of stay sutures were used to hold the margin of the atrial septum, were pulled out of the port, and secured properly. After the valve was assessed, mitral valve surgery and the tricuspid valve procedure were performed. After the heart was carefully de-aired and evaluated by TEE, cardiopulmonary bypass was terminated, and all incisions were closed.

Conventional open mitral valve surgery was performed via median sternotomy.

### Questionnaire survey

The assessments were initiated in the third month after surgery. The clinical parameters in the two groups included the surgical technique, postoperative morbidity, length of hospital stay and costs. Severe events were defined according to guidelines published by Akins [7]. The Chinese version of the MOS 36-Item Short-Form Health Survey(SF-36) was selected as the main clinical assessment tool for HRQoL, and two sets of questionnaires, including the Scar Cosmesis Assessment and Rating (SCAR) Scale and numerical rating scale (NRS), were used to evaluate pain intensity and the cosmetic appearance of the scar in all participants.

The MOS 36-Item Short-Form Health Survey is the most commonly used assessment tool for HRQoL [12]. We used the Chinese version of the SF-36 to assess health status. This SF-36 is suitable for assessing the health of clinical populations with various diseases. The questionnaire consists of 36 items and measures 8 health domains (general health, mental health, bodily pain, physical role, physical function, vitality, role emotional and social function) [13, 14]. A higher score for a given subscale suggests a higher QoL regarding that domain.

Some patients were illiterate and elderly and may have had visual and cognitive functional impairments. We used the NRS as a simple and valid alternative assessment of pain intensity [15, 16]. We used the 11-point numerical rating scale (NRS-11) for the assessment of pain intensity, where 0 = no pain and 10 = the most severe pain imaginable. The NRS-11 provides a sufficient level of discrimination for patients to describe pain intensity [17].

All the surgical wounds eventually developed into scars. Post-surgical scars with cosmetic issues cause functional and psychosocial impairments. The evaluation of postsurgical scar formation is very important. The SCAR scale is a valid and reliable scale for the assessment of postsurgical linear scars. The scale includes six questions with six parameters scored by a clinician (scar spread, erythema, dyspigmentation, suture marks, hypertrophy/atrophy, overall impression) and two questions requiring only a yes/no response from the patient [18, 19]. Scores can be determined by direct observation and evaluation or by using high-quality images. The patients’ responses to the questions may be either verbal or written [20].

### Statistical analysis

SPSS 22.0 was used as statistical software, and *P*-values < 0.05 were defined as statistically significant. The mean ± standard deviation was calculated for quantitative data with a normal distribution; for nonnormally distributed data, the Mann-Whitney U-test was used. We used the independent samples t-test or analysis of variance for continuous variables. For the categorical data, the χ2 test was applied. We used Spearman’s correlation coefficient for ranked data to assess the correlation between pain intensity or the SCAR scale scores and the SF-36 scores.

## Results

A total of 163 consecutive patients underwent mitral valve surgery (78 patients underwent the modified totally endoscopic approach (EA, *n* = 78), and 85 patients underwent the median sternotomy approach (SA, *n* = 85)) were included. There were no significant differences in the demographics or echocardiography findings between the two groups (Table 1). There was a trend towards fewer mitral valve repair (*p* = 0.13) and fewer tricuspid valve plasty (*p* = 0.10) procedures being performed in the EA group. The number of bioprosthetic valve replacements was significantly higher in the EA group (*p* = 0.04), whereas the subvalvular apparatus was preserved more often in the SA group (*p* = 0.01).

**Table 1:** Demographic and Intra-operative data compared between EA group and SA group

Item	EA group	SA group	*P*
Male/Female	43/35	55/30	0.21
Age (years)	51.49 ± 11.87	51.69 ± 11.69	0.91
Current NYHA (median)	II	II	
BMI (kg/m^2^)	22.66 ± 1.59	22.50 ± 1.80	0.55
Lesion types of mitral valve			
Mitral stenosis	26	21	0.23
Mitral insufficiency	38	40	
Mitral stenosis and insufficiency	14	24	
LVED	58.35 ± 8.32	57.72 ± 8.69	0.64
LVEF (%)	58.50 ± 6.94	57.42 ± 5.79	0.29
Surgery strategy			
Mitral valve repair	8	17	0.13
Mitral valve replacement	70	68	
bioprothetic valves	39	28	0.04
Preservation of Subvalvular Apparatus	33	54	0.01
tricuspid valve plasty	15	27	0.10

The postoperative complications are shown in Table 2. The rate of postoperative adverse events was similar between the two groups. Serious complications did not occur, and reoperations were not needed during the follow-up period. The length of intensive care and postoperative hospital stay did not statistically significantly differ between groups. One case of inguinal lymphatic leakage and a case of right femoral vein thrombosis were detected in patients after minimally invasive surgery. Immediately after the patient was diagnosed, she was transferred to the vascular surgery department for catheter-directed thrombolysis combined with stent placement for acute femoral vein thrombosis, and warfarin anticoagulation was continued.

**Table 2:** Postoperative Data

Item	EA group	SA group	*P*
Structural Valve Deterioration	0	0	NS
Nonstructural Dysfunction	0	0	NS
Valve Thrombosis	0	0	NS
Embolism	1	0	NS
Bleeding Event	2	1	0.94
Operated Valve Endocarditis	0	0	NS
Reintervention	0	0	NS
Conduction Disturbance	0	0	NS
Poor wound healing	2	2	1.00
Pneumothorax	3	0	0.21
Subcutaneous emphysema	2	0	0.45
LVEF (%)	54.98 ± 7.38	56.38 ± 5.22	0.16
LVED (mm)	55.76 ± 6.80	54.83 ± 6.19	0.36
ICU stay (days)	2.10 ± 1.12	2.17 ± 0.82	0.16
Current NYHA (median)	I	I	
Postoperative hospital stay (days)	5.17 ± 1.75	5.93 ± 1.14	0.16
Hospital costs (RMB)	100,980.24 ± 7405.36	101,309.91 ± 6911.20	0.77

The patients were followed up in the 3rd month after the operation. We assessed HRQoL in the two groups using the MOS SF-36, which demonstrated significant differences in the scores for the bodily pain and mental health subscales (Table 3). The NRS scores and the SCAR scores were evaluated in both groups (Table 4). The difference in the SCAR scores between the two groups was significant, with the EA group being more satisfied with the aesthetic appearance of the incision (P<0.05). There were significantly fewer complaints of postoperative pain in the EA group than in the SA group. The scores were significantly lower (*P* < 0.05). Evaluation on pain intensity and aesthetics showed a significant correlation (P < 0.05) with the SF-36 subscale evaluation results. The rank correlation coefficient between the SF-36 scores and the NRS and SCAR scores is shown in Table 5. The high correlation coefficient and low *p*-value indicate that the correlation is highly significant. The correlation coefficient between the score of the bodily pain subscale of the SF-36 and the NRS score was − 0.819, with a p-value less than 0.001 (see Table 5). The correlation coefficient between the score of the mental health subscale of the SF-36 and the SCAR scale score was − 0.791, with a p-value less than 0.001 (see Table 5). We observed that bodily pain was strongly correlated with the NRS score. Moreover, the mental health score was correlated with the SCAR scores. These findings indicate that patients who obtained a higher score on the NRS demonstrated lower SF-36 scores on the bodily pain subscale and those who obtained a higher score on the SCAR scale demonstrated lower SF-36 scores on the mental health subscale.

**Table 3:** SF-36 scores were compared between the two groups at 3 months after surgery

Item	EA group	SA group	*P*
Physical functioning	77.750 ± 8.0774	77.462 ± 8.0827	0.82
Role physical	71.15 ± 15.12	68.82 ± 15.39	0.33
Bodily pain	77.05 ± 14.78	70.12 ± 12.58	0.001
General health	65.13 ± 13.31	63.29 ± 1.51	0.347
Vitality	64.17 ± 11.99	63.29 ± 11.51	0.64
Social functioning	71.71 ± 12.20	70.38 ± 11.87	0.48
Role emotional	65.14 ± 17.86	62.18 ± 13.97	0.24
Mental health	74.62 ± 13.63	68.42 ± 17.95	0.015

**Table 4:** assessment of different pain intensity and scar scale

Item	EA group	SA group	*P*
NRS pain score	0.40 ± 0.67	1.24 ± 0.65	*p* < 0.01
SCAR score	0.64 ± 0.60	1.75 ± 0.77	*p* < 0.01

**Table 5:** The coefficient of rank correlation between the SF-36 scores and the NRS and SCAR scale scores

scale	Coefficient of rank correlation	Coefficient of rank correlation	*P*1 value	*P*2 value
Physical functioning	0.015	−0.042	0.848	0.597
Role physical	−0.029	−0.032	0.709	0.681
Bodily pain	−0.819	− 0.181	*p* < 0.001	0.021
General health	−0.317	− 0.072	*p* < 0.001	0.355
Vitality	−0.283	− 0.035	*p* < 0.001	0.660
Social functioning	−0.167	0.042	0.033	0.591
Role emotional	−0.232	−0.145	0.003	0.065
Mental health	−0.132	−0.791	0.092	*p* < 0.001

P1: The coefficient of rank correlation between the SF-36 subscale scores and the NRS scores

P2: The coefficient of rank correlation between the SF-36 subscale scores and the SCAR scale scores

Correlation coefficients: 0–0.20 = “week”; 0.21–0.40 = “fair”; 0.41–0.60 = “moderate”; 0.61–0.80 = “strong”; 0.81–1.00 = “strongly correlation”

Table 3 shows that the EA group had higher scores on the bodily pain and mental health subscales than did the SA group, and a higher score represents less bodily pain and better mental health. Table 4 shows that the EA group had lower total scores on the NRS and SCAR scales than did the SA group, and a lower score represents less severe pain and a better cosmetic appearance. According to the relationship shown in Table 5, bodily pain was strongly correlated with the NRS score. Moreover, the mental health score was correlated with the SCAR scores. The EA group achieved a reduction in pain and better aesthetic outcomes, and the related HRQoL in the EA group was better than that in the SA group.

## Discussion

As several literature reviews have mentioned, MIMVS has been successfully performed with techniques modified over the past twenty years and has recently been proven to be a safe and effective treatment [21, 22]. Compared to conventional approaches, MIMVS is safe and yields similar results. Reviewing all the studies on mortality with MIMVS, the majority of studies showed no difference between the minimally invasive approach and the median sternotomy approach [23, 24]. Many authors have shown that the morbidity and mortality rates of MIMVS are similar to those of the traditional sternotomy approach for mitral valve surgery but that MIMVS yields a shorter recovery time, shorter hospital stay, less severe pain and better cosmesis [21, 24, 25]. With the desire to reduce the mortality and morbidity rates associated with mitral valve surgery, the minimally invasive techniques have continued to evolve and achieve excellent results. MIMVS has equally good outcomes as well as shorter hospital stays and better resource utilization [26, 27].

Different from common totally endoscopic approach, we performed mitral valve surgery with a modified, minimally invasive, totally endoscopic approach. Cardiopulmonary bypass was established only through the femoral artery and femoral vein with a single two-stage femoral venous cannula. This technique avoids the need for the insertion of a right internal jugular vein cannula, which can lead to many complications, such as bleeding, pneumothorax, carotid artery injury and haematoma. In addition, the time required to prepare the patient preoperatively is greatly reduced because jugular vein cannulation is not needed. Although some individuals may argue that air may be entrained in the venous cannula when the right atrium is open, this issue can be safely avoided if the two perforated segments are positioned correctly. Another issue may be that the non-perforated section of the cannula crosses the right atrium, which may limit exposure of the heart valve. However, as shown in Fig. 3, the non-perforated segment of the cannula is located at the septum and does not obstruct the valve view. In conclusion, a single two-stage cannula can be safely used during surgery in the right atrium, and it allows the pump to function properly when the left atrium is retracted during mitral valve surgery or when the right atrium is opened during tricuspid valve surgery. In fact, it is our preferred method of venous return during totally endoscopic surgery.

In our study, the morbidity rates after cardiac valve interventions in the two groups were similar. No cases of structural valve deterioration or valve thrombosis were observed in either group. There were no differences in major adverse events, such as reoperations for bleeding, operated valve endocarditis, or reintervention. In addition, we found that for the cost for total endoscopic mitral valve surgery is similar to that of median sternotomy mitral valve surgery (100,980.24 vs 101,309.91 RMB, p>0.05). Although previous study shows that it can reduce intensive care unit days and postoperative hospital stays, this technique is accompanied by appreciable costs for medical consumables. Consumables such as HTK solution, femoral artery and femoral vena cava cannula were associated with a significant increase in medical costs [26, 28].

Although the mortality and morbidity after totally endoscopic mitral valve surgery have been reported in previous studies, the effect of totally endoscopic mitral valve surgery on HRQoL has rarely been studied, especially in the Chinese population. HRQoL may be influenced by factors such as the mental state of the patient, the pain intensity and even the patient’s recognition of cosmesis. Operation safety and postoperative outcomes of totally endoscopic mitral valve surgery and median sternotomy mitral valve surgery have been proven to be equivalent. Thus, the effect of totally endoscopic mitral valve surgery on HRQoL should be considered when the surgical approach is evaluated and selected.

In this study, we aimed to compare the effect of the totally endoscopic approach and that of the median sternotomy approach on the HRQoL of patients who had undergone mitral valve surgery. We also focused on the effect of two different approaches with respect to pain intensity, cosmetic appearance, and the correlations between pain intensity and cosmetic appearance and HRQoL in patients. In our literature search, we could not find any studies that compared HRQoL in patients undergoing mitral valve surgery with different surgical approaches. Moreover, no studies compared pain intensity and cosmetic appearance between the two surgical approaches, and no studies assessed the impacts of pain intensity and cosmetic appearance on quality of life. We assumed that totally endoscopic mitral valve surgery and median sternotomy mitral valve surgery have similar impacts on the HRQoL of patients.

All patients in the study completed the SF-36 and provided information on the pain intensity and the SCAR scale. We used the Chinese version of the SF-36 to assess HRQoL. We found that the SF-36 scores of the minimally invasive group were superior to those of the median group in the two subscales. We detected significant differences in bodily pain and mental health between the totally endoscopic approach group and the median sternotomy approach group. Moreover, the scores on the other six subscales (including physical functioning, general healthy, role physical, vitality, social role functioning, and emotional role functioning) were higher in the totally endoscopic approach group than in the median sternotomy approach group.

The impact of pain intensity and cosmetic appearance on a patient’s health-related quality of life, different from severe complications, is often underestimated by surgeons. R.P. Alston reported that chronic post-sternotomy pain occurs in 40–50% of patients. Of these patients, 33–66% had pain lasting more than 3 months [29]. Pain after cardiac surgery is still underestimated and can be a problem. J Meyerson also reported that 28% of patients who underwent median sternotomy for cardiac surgery could suffer from non-cardiac pain. Mild pain was present in the majority of patients, and severe pain was present in 1% of patients [30]. Chronic pain associated with the sternotomy incision is a well-recognized complication that severely impacts patients’ daily life [31]. Chronic pain usually has a negative influence on mood and can impair patients’ ability to perform activities [29]. In a study that investigated persistent pain after cardiac surgery, 7% of the 244 patients reported experiencing interference with everyday life [32].

The cause of persistent post-sternotomy pain includes rib fractures, scars, tissue damage, steel wire sutures, intercostal nerve trauma, and infections in sternal and sternal dehiscence [33]. This totally endoscopic incision, which avoids dividing the sternum and cracking of the ribs, may reduce patient distress and pain. Our study proved that the postoperative pain intensity associated with EA was different from that associated with SA, and the pain intensity of the EA group was significantly lower than that of the SA group. This result is consistent with those in previous studies [22, 24, 34].

Another apparent advantage of totally endoscopic mitral valve surgery compared with the median sternotomy approach is the resulting cosmetic appearance. In this study, we also compared the SCAR scores of the two groups, and our study proved that the SCAR scores of the EA group were significantly better than those of the SA group.

The coefficient of rank correlation between the SF-36 scores and the pain intensity and the SCAR scores indicate that bodily pain was closely related to pain intensity (NRS scores) and that mental health was closely related to scar aesthetics (the SCAR scale scores).

According to the data summarized above, the EA group had a better impact on HRQoL, as well as mild pain and a better cosmetic appearance than did the SA group, leading to a better impact on HRQoL. The postoperative complications were similar between the two groups. Therefore, totally endoscopic mitral valve surgery can be performed as an alternative surgery in China, as it does not yield any significant differences in related postoperative complications but does yield significant differences in pain intensity, cosmetic appearance and HRQoL.

This study has some limitations. First, this was a retrospective study conducted in a single institution in China, and selection and recall bias may contribute to the findings. Second, the cohort was small, and the follow-up period was short. Despite these limitations, we still believe that this study has some significance.

## Conclusions

The results of this study showed that modified totally endoscopic mitral valve surgery has an equally treatment outcome as does traditional median sternotomy mitral valve surgery. The totally endoscopic approach mitral valve surgery has higher scores on the bodily pain and mental health subscales of the SF-36 than did median sternotomy, besides, the endoscopic approach group had lower total scores on the NRS and SCAR scales than did the median sternotomy group, indicating the totally endoscopic mitral valve surgery can achieve reductions in pain and better aesthetic outcomes, and the related HRQoL in the totally endoscopic approach group was better than that of the median sternotomy approach group. Our study suggested that the totally endoscopic approach was superior to the median sternotomy in terms of pain intensity, aesthetic appearance and health-related quality of life. It is recommended that the type of approach to be used for mitral valve surgery is selected in each centre on the basis of the actual situation. Additional studies with longer follow-up periods are recommended to assess the HRQoL of patients who undergo these surgeries with these two different approaches.

## Data Availability

Data sharing not applicable to this article as no data sets were generated or analyzed during the current study.

## Abbreviations

HRQoL: Health-related quality of life
SCAR: Scar Cosmesis Assessment and Rating
NRS: Numerical Rating Scale
MIMVS: Minimally invasive mitral valve surgery
TEE: Transesophageal echocardiography
SF-36: The MOS 36-Item Short-Form Health Survey
NYHA class: New York Heart Association functional classification
BMI: Body mass index
LVED: Left ventricular end diastolic
LVEF: Left ventricular ejection fraction
